# Effects of Freshwater Pollution on the Genetics of Zebra Mussels (*Dreissena polymorpha*) at the Molecular and Population Level

**DOI:** 10.1155/2014/795481

**Published:** 2014-04-27

**Authors:** Emilia G. Thomas, Maja Šrut, Anamaria Štambuk, Göran I. V. Klobučar, Alfred Seitz, Eva Maria Griebeler

**Affiliations:** ^1^Department of Ecology, Zoological Institute, University of Mainz, P.O. Box 3980, 55099 Mainz, Germany; ^2^Department of Zoology, Faculty of Science, University of Zagreb, Rooseveltov Trg 6, 10000 Zagreb, Croatia

## Abstract

Revealing long-term effects of contaminants on the genetic structure of organisms inhabiting polluted environments should encompass analyses at the population, molecular, and cellular level. Following this concept, we studied the genetic constitution of zebra mussel populations from a polluted (Dp) and reference sites (Cl) at the river Drava, Croatia, and applied microsatellite and DNA damage analyses (Comet assay, micronucleus test (MNT)). Additionally, mussels from both populations were exposed to polluted wastewater in the laboratory for three days, and DNA damage was analyzed to evaluate acclimatization and genetic adaptation of the investigated populations to the polluted environment. The two populations differed in their genetic constitution. Microsatellite analysis suggested that Dp had undergone a genetic bottleneck. Comet assay did not indicate any difference in DNA damage between the two populations, but MNT revealed that Dp had an increased percentage of micronuclei in hemocytes in comparison to Cl. The laboratory experiment revealed that Dp had a lower percentage of tail DNA and a higher percentage of micronuclei than Cl. These differences between populations were possibly caused by an overall decreased fitness of Dp due to genetic drift and by an enhanced DNA repair mechanism due to acclimatization to pollution in the source habitat.

## 1. Introduction

The fast growing human population has altered freshwater ecosystems profoundly [1]. Hazardous substances present in the wastewater from agriculture, industry, and human settlements end up in aquatic ecosystems [2]. Effluents of wastewater treatment plants (WWTP) are one of the major sources of genotoxicants in surface waters [3, 4]. Evolutionary toxicology investigates the effects of chemical pollutants on the genetics of natural populations [5–7]. Toxicants may induce DNA damage along with long-term DNA changes in freshwater organisms. It has been suggested that the resulting genomic instability plays an important role in decreasing fitness of populations and hence may have significant consequences for short- and long-term survival of populations [2]. Moreover, pollution induces stochastic effects on population genetics causing a decline in population size and consequently inbreeding and the overall loss of genetic diversity. These genetic processes can reduce overall population fitness and accelerate population extinction [8–10].

To obtain an integrated assessment of the impact of pollution on populations, the use of multiple biomarkers at different levels of biological organization has been strongly suggested [2, 7, 11]. If chemical contamination is responsible for an emergent effect at the population level, responses at lower levels of biological organization should also be apparent [11]. The establishment of this causal relationship is essential to support the conclusion that an emerging population effect is due to contamination exposure [7].

The zebra mussel* Dreissena polymorpha *has been applied as bioindicator for passive as well as active biomonitoring [12–14], for example, for genotoxicity monitoring in freshwater ecosystems [15].

In particular, we present a case study of two mussel populations from the river Drava (Croatia). These two populations are considered to have the same genetic background but are exposed to different environmental conditions. The first population (Dp) was collected at a polluted site where mussels were constantly exposed to the effluent of a municipal WWTP, whereas the second (Cl) was collected at a reference site.

We first studied the genetic constitution of both populations applying microsatellite analysis at the population level and DNA damage analysis at the molecular and cellular level. For the population level we hypothesized to detect a genetic bottleneck in population Dp, caused by increased mortality resulting from the exposure of the population to the WWTP effluent (hypothesis 1.1). This bottleneck could be a result of selection or of random genetic drift due to a decrease in population size. At the molecular and cellular level, we expected increased genotoxic effects in Dp compared to Cl (hypothesis 1.2).

Second, in a laboratory experiment, we exposed randomly selected individuals from both populations to polluted municipal wastewater. For this experiment, we expected that the two populations would react differently to these altered environmental conditions. (i) Under the assumption that selective processes have changed the genetic constitution of population Dp in the source habitat, Dp could be more resistant to the wastewater and show less DNA damage than Cl (hypothesis 2.1). (ii) If alternatively, Dp had a decreased genetic diversity due to random genetic drift leading to a decreased fitness under altered environmental conditions [10, 16], we expected that Dp would be more severely affected by the wastewater and exhibit a higher level of genotoxic effects than the control population Cl (hypothesis 2.2).

## 2. Materials and Methods

### 2.1. Sampling Sites

**Figure 1:**
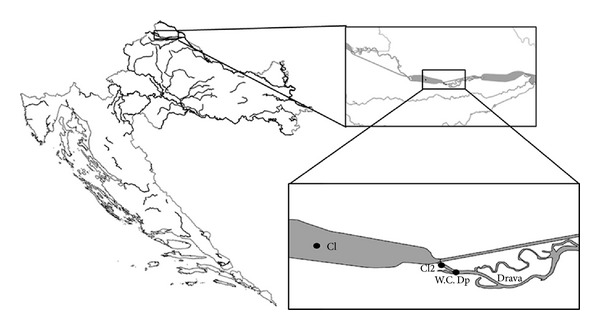
Location of the sampling sites in Croatia. Cl: Čakovec lake (control population); Cl2: reference site for water analyses and reference sampling site of mussels used in the laboratory experiment; Dp: contaminated population located downstream from the mouth of the wastewater treatment plant effluent channel (W.C.). For sediment analyses a sample was taken in the W.C.

The zebra mussels were sampled in July and August 2009 in northern Croatia (Figure 1). The reference site Cl (46.3143678°N, 16.4154166°E) was situated in the Čakovec lake. Due to the high water level at the time point of water sampling, water samples and mussels for the laboratory experiment were taken at the second reference site situated just behind the dam of the Čakovec lake, about 600 m upstream from Dp (46.306274°N, 16.471809°E). The contaminated sampling site (Dp: 46.303452°N, 16.478352°E) was about 750 m downstream from Cl. Approximately 10 m upstream from the Dp site, a municipal WWTP effluent flows into the river Drava.

### 2.2. Water and Sediment Analyses

All chemical analyses of water and sediment were performed by the Institute of Public Health “Dr. Andrija Štampar,” Zagreb, Croatia, except for pH and oxygen, which were directly measured in the field.

#### 2.2.1. Water Analyses

Water samples were collected at the Dp site, at the WWTP effluent channel, approximately 20 m upstream from the outlet into the river Drava, and at a reference site Cl2 (Figure 1). For the laboratory experiment, we additionally sampled water in a wastewater channel leading to the main city collector of municipal wastewater in Zagreb. In the water samples, pH and the concentrations of dissolved oxygen, chromium, copper, nickel, lead, iron, cadmium, zinc, manganese, twelve polycyclic aromatic hydrocarbons (PAHs), and polychlorinated biphenyls (PCBs) were measured. The pH was measured with a pH meter (type WTW pH 526). Dissolved oxygen was measured with an oximeter (type WTW OXI 730). The concentrations of metals (Cr, Cu, Ni, Pb, Fe, Cd, Zn, and Mn) in the water were determined using inductively coupled plasma-optical emission spectrometry (ICP-OES, IRIS Intrepid II XSP, Thermo) according to the HRN EN ISO 11885 [17] standard method. PAHs were determined by a high performance liquid chromatography (HPLC system Agilent 1100 Series-thermostatted autosampler G1329A, binary pump G1312A) with fluorimetric detection (fluorescence detector G1321A by Agilent Technologies) (HPLC-FLD). The 12 PAHs (naphthalene, fluorene, phenanthrene, anthracene, fluoranthene, pyrene, chrysene, benzo(*b*)fluoranthene, benzo(*k*)fluoranthene, benzo(*a*)pyrene, benzo(*g,h,i*)perylene, and indeno(1,2,3-*cd*)pyrene) were analyzed according to the EPA 550 [18] standard method. The total PAH concentration corresponded to the sum of the concentrations of the 12 PAHs analyzed. For PCBs, water samples were extracted by dichloromethane. The extracts were evaporated on a rotary evaporator (Rotavapor R-210 with Heating Bath B-491, Vacuum Pump V-700, and Vacuum Controller V-850, Büchi), purged, and then concentrated in a stream of nitrogen gas. PCBs were quantified by a gas chromatography with a ^63^Ni Electron capture detector (GC-17A-ECD, Shimadzu) according to the HRN EN ISO 6468 [19] standard method.

#### 2.2.2. Sediment Analyses

Only one sediment sample could be collected in the WWTP effluent channel (W.C., Figure 1), since the bottom at Cl2 (Figure 1) and Dp consisted of small pebbles. To evaluate the degree of pollution of this sediment sample we measured the concentrations of heavy metals (chromium, copper, nickel, lead, cadmium, zinc, cobalt, and mercury), arsenic, molybdenum, mineral-oil hydrocarbons, PAHs, and PCBs.

The methods used for determining heavy metal concentrations (Cr, Cu, Ni, Pb, Cd, Zn, Co, and Hg), arsenic, mineral oil hydrocarbons, and PAHs have been previously described in detail [20]. The concentration of Mo was determined in the same way as the heavy metals. For PCB detection, the oven-dried samples (5 g) were transferred to a Soxhlet apparatus and extracted with 100 mL of n-hexane. The extracts were evaporated on a rotary evaporator (Rotavapor R-210 with Heating Bath B-491, Vacuum Pump V-700, and Vacuum Controller V-850, Büchi) and concentrated in a stream of nitrogen gas. They were then transferred into a centrifuge tube and cleaned up with concentrated sulphuric acid (min. 96%). If the samples were highly contaminated repeated acid clean-up was employed. PCBs were quantified by a gas chromatography with a ^63^Ni Electron capture detector (GC-17A-ECD, Shimadzu) according to EPA 8082 [21] standard method.

### 2.3. Microsatellite Analysis

For population genetic analyses, the sample size was 48 individuals for each of the populations. Genomic DNA was extracted from each of the individuals from the posterior adductor muscle of ethanol-preserved samples using the High Pure PCR Template Preparation Kit (Roche Diagnostics GmbH, Mannheim, Germany). The six primers used for microsatellite analysis and the respective PCR protocols are based on Naish and Boulding [22], Astanei et al. [23], and Thomas et al. [24]. The primers A6, B6, B9, and C5 from Naish and Boulding [22] and Astanei et al. [23] were partially modified in the Institute of Zoology, University of Mainz (Table 1) using the program PRIMER3PLUS [25], or by PIG-tailing [26]. The primers Dpol9 and Dpol19 were those from Thomas et al. [24]. The genotype data were generated on ABI 3130 xl DNA Analyzer. For each of the individuals the allele fragment size calls were made using the GENEMAPPER 4.0 software (Applied Biosystems, Foster City, CA, USA). One individual of population Dp provided a low DNA quality and was therefore removed from the genetic data set.

The microsatellite data set was checked for scoring errors due to stuttering, large allele dropouts, and null alleles with the program Microchecker [27]. We tested the potential influence of scoring errors on Hardy-Weinberg Equilibrium (HWE) with the jackknife procedure described by Morin et al. [28] and executed by the program “R” [29]. Linkage disequilibria between pairs of loci were tested using the program GENEPOP, version 4.0.10 [30].

### 2.4. Comet Assay

Mussels collected from the two sites Cl and Dp were transported within 90 min to the Department of Zoology, Faculty of Science in Zagreb. Hemolymph was withdrawn from the posterior adductor muscle sinus with a hypodermic syringe for subsequent Comet assay and MNT. The Comet assay was conducted as described in detail by Štambuk et al. [31]. For each individual, one slide with 50 cells was examined, and the extent of DNA migration was determined as a percentage of the tail DNA using an image analysis system Komet 5, Kinetic Ltd.

### 2.5. Micronucleus Test

Aliquots of 0.1 mL hemolymph mixed with 0.1 mL phosphate-buffered saline (PBS) in 10 mM ethylenediaminetetraacetic acid (EDTA) were placed on slides and left for 15 min in a humidified chamber at room temperature allowing hemocytes to settle down. The subsequent procedure was conducted as described previously [31]. Micronuclei were identified according to the criteria given in Majone et al. [32] and Kirsch-Volders et al. [33].

### 2.6. Statistical Analyses

#### 2.6.1. Field Populations—Genetic Bottlenecks

We checked both field populations for possible bottlenecks using the program BOTTLENECK, version 1.2.02 [34]. With this program, the population sample is tested for an excess of heterozygosity in comparison to the heterozygosity expected at mutation-drift equilibrium (*H*_*E*_ > *H*_EQ_). In a bottlenecked population, the number of alleles is reduced faster than the allele frequencies by a strong reduction in population density. As *H*_*E*_ is calculated from allele frequencies and *H*_EQ_ is calculated from allele numbers, a significant excess of heterozygosity (*H*_*E*_ > *H*_EQ_) is evidence of a recent bottleneck in a population [35]. We chose the two-phase mutation model (TPM) in this analysis, since it has been described as the most appropriate model for microsatellite loci [35, 36]. The Wilcoxon test was used to test statistical significance of bottlenecks.

#### 2.6.2. Field Populations—Genetic Diversity and Hardy-Weinberg Equilibrium (HWE)

The overall observed and expected heterozygosity (genetic diversity) of the two populations and respective heterozygosities for each of the loci were estimated with the program GENEPOP, version 4.0.10 [30]. The same program was applied for testing overall HWE by the exact “HW test” for each population and by the statistically more powerful score test (*U* test) differentiating between heterozygote deficiency (*H*_*O*_ < *H*_*E*_) and excess (*H*_*O*_ > *H*_*E*_) [37]. In case of overall deficiency of heterozygotes the score test of heterozygote deficiency was also applied to each of the six loci to detect which of the loci had caused a deficiency of heterozygotes in the populations Cl and Dp. In all score tests conducted, Markov chain parameters were 10,000 for dememorization, with 500 batches and 5,000 iterations per batch.

#### 2.6.3. Field Populations—Population Differentiation and Inbreeding

To assess overall differences in gene pools of the populations Cl and Dp, we calculated the differentiation between the two populations (*F*_ST_) and the inbreeding coefficients (*F*_IS_) using the *F*-statistics by Weir and Cockerham [38] as implemented in the program FSTAT [39]. Weir and Cockerham [38] weight allele frequencies according to sample sizes, as well as the estimates of these statistics, are not influenced by differences in sample sizes of populations. The *F*_ST_ and *F*_IS_ estimates were calculated over all loci, as well as separately for each of the loci.

#### 2.6.4. Field Populations—Analysis of Molecular Variance (AMOVA)

To rate changes in the genetic composition of the field populations between generations, the individuals of both populations were divided into three distinct age classes. We determined the age of individuals by counting the annual rings on the surface of their shells [40]. The first age class consisted of one- and two-year-old zebra mussels, the second age class contained three- and four-year-old mussels, and the third age class comprised the five-and-six-year-old mussels.

The *F*-statistics of the age classes (across all six loci) were estimated and tested for significant deviation from zero, using a nonparametric permutation approach described by Excoffier et al. [41]. Furthermore, the age-class-specific inbreeding coefficients (*F*_IS_) were estimated and also tested for significance.

The distance method applied in the locus-by-locus AMOVA was the number of different alleles. With the help of this method, the locus-specific inbreeding coefficients (*F*_IS_), variances among age classes within populations (*F*_SC_), variances among age classes among populations (*F*_CT_), and the overall fixation indices (*F*_IT_) were calculated and tested for significant deviation from zero. All AMOVA analyses were computed with the program Arlequin, version 3.5.1.2 [42].

#### 2.6.5. Comet Assay and MNT

For each group (field and laboratory populations, see below), mean values of DNA damage were calculated based on the mean of each individual within a group. The data are presented as mean ± SEM (standard error), for both Comet assay and MNT. To assess differences between groups, we performed the Mann-Whitney *U* test.

### 2.7. Laboratory Exposure

Approximately 200 mussels per site were acclimatized to laboratory conditions for two weeks in glass aquaria. The aquaria contained dechlorinated, well-aerated water, which was renewed every other day, but not any sediment. Every other day the mussels were fed with algae* Chlorella *sp.

After this acclimatization period, 50 mussels of each population were exposed to the polluted municipal wastewater of Zagreb in aquaria for three days. The wastewater in the aquaria was renewed every day. At the same time, other samples of 50 mussels of each population were exposed to dechlorinated tap water and were used as a control. At the end of the experiment, seven to twelve individuals were taken from each treatment to assess genotoxicity by the Comet assay and MNT.

## 3. Results

### 3.1. Water and Sediment Analysis

Results of the water and sediment chemical analysis are presented in Table 2. Very slight differences were observed between the water parameters at the sampling sites in the WWTP effluent channel (WcW) and Dp in comparison to the clean site Cl2 (Figure 1). Only decreased oxygen concentration clearly indicated increased water pollution at these two polluted sites.

The sediment analysis of the WWTP effluent channel clearly showed that the water running through this channel has been heavily polluted, at least from time to time.

### 3.2. Field Populations—Review of Microsatellite Data

The analysis of the genetic data set with the program Microchecker [27] did not detect any scoring errors due to stuttering or large allele dropouts. It also did not reveal any hint for existing null alleles, except for locus C5 in Dp. This locus had a significant excess of homozygotes, which could indicate the presence of null alleles. The estimated rate of possible null alleles of locus C5 in Dp was low (0.0723). The six individuals that were suspected to have null alleles by the software Microchecker were not considered to be influential on HWE estimates for the population based on the jackknife procedure [28]. Therefore, we did not remove these individuals from the data set. Moreover, low null allele frequencies have a negligible impact even in parentage analysis, whereupon parentage analysis is expected to have a higher bias from null alleles than analysis of population structure [43]. We detected no significant linkage disequilibrium between all pairs of the six loci studied applying sequential Bonferroni correction.

### 3.3. Field Populations—Genetic Bottlenecks

The Wilcoxon test revealed a significant excess of heterozygosity (*H*_*E*_ > *H*_EQ_) in Dp (*P* = 0.023), but not in Cl (*P* = 0.219). This result suggests that the Dp population has undergone a recent genetic bottleneck [35]. Furthermore, there was a loss of rare alleles in population Dp at four out of the six analyzed loci.

### 3.4. Field Populations—Population-Specific Genetic Diversity and HWE

The genetic diversity computed over all loci by GENEPOP was similar for both populations with *H*_*E*_ = 0.815 in Cl and *H*_*E*_ = 0.807 in Dp. Based on all loci, the observed heterozygosity *H*_*O*_ was 0.809 in Cl and 0.760 in Dp. *H*_*O*_ deviated significantly from *H*_*E*_ only for Dp, but not for Cl (Table 3). *H*_*O*_ was lower in Dp than in Cl for all six analyzed loci. For Dp, locus C5 showed the strongest difference between observed and expected heterozygosity (*H*_*O*_ = 0.638, *H*_*E*_ = 0.765) while no difference was observed for this locus in Cl (*H*_*O*_ = 0.750, *H*_*E*_ = 0.784) (Table 3).

The exact tests implemented in GENEPOP did not show any significant deviation from HWE for both populations (*P*_Cl_ = 0.443,  *P*_Dp_ = 0.237). The score tests of heterozygote excess also corroborated HWE (*P*_Cl_ = 0.586, *P*_Dp_ = 0.967), whereas the score tests of heterozygote deficiency revealed a significant deficit of heterozygotes in Dp (*P* = 0.033), but not in Cl (*P* = 0.414). Score tests for heterozygote deficiency individually carried out for each of the six loci and each of the two populations revealed that all loci conformed to HWE, except for locus C5 in Dp, which had a significant deficit of heterozygotes (*P* = 0.013, Table 3).

### 3.5. Field Populations—Population Differentiation

The score test indicated a significant deficit of heterozygotes in Dp. The largest *F*_IS_ value was observed for locus C5 in population Dp (*F*_IS_ = 0.167, Table 3). The fixation index (*F*_ST_ = −0.001) of the two populations did not differ from zero and thus indicated no genetic differentiation between the two populations (Table 3).

### 3.6. Field Populations—AMOVA of the Different Age Classes in Cl and Dp and Locus-by-Locus AMOVA

AMOVA indicated that the major proportion of genetic variation was due to genetic differences within individuals (96.96%). The overall inbreeding coefficient (*F*_IS_ = 0.032, *P* = 0.044) and the overall fixation index (*F*_IT_ = 0.030, *P* = 0.043) both deviated significantly from zero. In contrast, the variances among age classes within the populations (*F*_SC_ = −0.001, *P* = 0.542) and among the populations (*F*_CT_ = −0.001, *P* = 0.556) were not significant (Table 4). The AMOVA analysis for the age classes of each of the two populations revealed that the second and third age classes of population Dp had significant *F*_IS_ values above zero, whereas all other age classes had insignificant *F*_IS_ values close to zero. This observation across loci demonstrated that the second age class (*F*_IS_ = 0.073, *P* = 0.036) and third age class (*F*_IS_ = 0.098, *P* = 0.035) of Dp had an increased proportion of homozygotes in comparison to the first age class of Dp and to all three age classes of Cl.

Locus-by-locus AMOVA carried out over all age classes showed that only locus C5 was significantly different from zero in the *F*-statistics. The overall fixation index was significant (*F*_IT_ = 0.094, *P* = 0.047) for locus C5 (Table 5).

### 3.7. Field Populations—Comet Assay and Micronucleus Test

**Figure 2:**
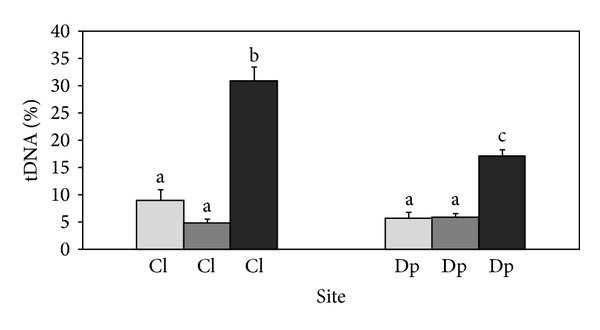
DNA damage measured by the Comet assay in the hemocytes of zebra mussels of the Cl and Dp populations, river Drava. Light grey bars correspond to field populations, dark grey bars to population samples exposed to dechlorinated tap water in the laboratory experiment, and black bars to population samples exposed to wastewater in the laboratory experiment. Different letters indicate statistically different DNA damage (*P* < 0.05).

Results of the Comet assay carried out for field populations are shown in Figure 2. The percentages of tail DNA did not differ significantly between the Cl and Dp sites. The frequency of cells exceeding 50% of tail DNA was 0.29% for the control population Cl and 0.30% for population Dp.

**Figure 3:**
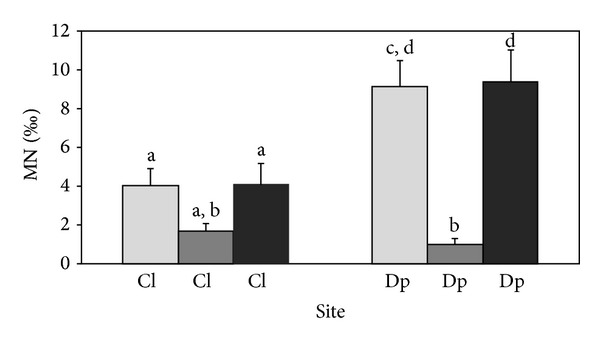
Number (per mill) of micronuclei (MN) in hemocytes of zebra mussels of the Cl and Dp populations, river Drava. Light grey bars indicate field populations, dark grey bars population samples exposed to dechlorinated tap water in the laboratory experiment, and black bars population samples exposed to wastewater in the laboratory experiment. Different letters indicate statistically different numbers of micronuclei (*P* < 0.05).

DNA damage as assessed by the MNT showed a significantly increased frequency of micronuclei in hemocytes for field mussels collected at the Dp site in comparison to the mussels originating from the Cl site (*P* = 0.008, Figure 3).

### 3.8. Laboratory Exposure—Comet Assay and Micronucleus Test

After two weeks of acclimatization and three days of exposure to dechlorinated tap water DNA damage reached basal levels that were very similar between the two populations, in both Comet assay and MNT. The Comet assay revealed a significantly higher DNA damage in hemocytes of mussels after the exposure to municipal wastewater than in the mussels of the control populations exposed to the dechlorinated tap water (*P*_Cl_ = 0.0002, *P*_Dp_ = 0.0002). The genotoxic response measured by the Comet assay in the individuals from the Cl population exposed to the wastewater was significantly higher in comparison to the response of mussels from the Dp population (*P* = 0.012, Figure 2). The frequency of cells exceeding 50% of tail DNA for the Cl population was 0% for the mussels in dechlorinated tap water and 10.99% for the mussels exposed to the wastewater. For the Dp population it was 0% for the mussels in dechlorinated tap water and 1.77% for the mussels exposed to the wastewater.

A significant increase in frequency of micronucleated hemocytes was detected in the mussels from the Dp population after the exposure to wastewater in comparison to the mussels in dechlorinated tap water (*P* = 0.0002, Figure 3), and also in comparison to the mussels from the Cl population and exposed to the wastewater (*P* = 0.023). The frequency of micronuclei in mussels from the Cl population exposed to the wastewater was not significantly different from the Cl mussels in tap water (*P* = 0.089).

There was no mortality of the mussels exposed to dechlorinated tap water during the two weeks of acclimatization or during the three days of exposure to the tap water. After the third day of exposure to municipal wastewater, the mortality rate was 77% for population Cl and 84% for population Dp.

DNA damage measured by the Comet assay and MNT did not differ significantly between the control laboratory population Cl (in tap water) and the control field population Cl (Comet assay: *P* = 0.118, MNT: *P* = 0.076, Figures 2 and 3).

## 4. Discussion

### 4.1. Field Populations—Population Level

#### 4.1.1. Genetic Bottleneck

Consistent with hypothesis 1.1 we found that Dp had undergone a significant genetic bottleneck which reflects a strong reduction in number of individuals in the past. The genetic bottleneck in Dp was accompanied by a loss of rare alleles in four out of six of the analyzed microsatellite loci. This suggests that nonselective random genetic drift had affected population Dp [44, 45]. Based on the observed high values of several analyzed heavy metals, PAHs, and mineral oil hydrocarbons in the sediment, we can conclude that this site is exposed to contamination inflow, and that the bottleneck in population Dp had probably resulted from the WWTP effluent contamination [46]. Genetic bottlenecks have also been observed for other populations of bivalves, for example, for razor clams (*Ensis siliqua*) after an oil spillage [47].

The water analysis at the Dp site and the effluent channel revealed only a decrease in oxygen concentration. However, in high flow systems like rivers, assessment of pollution status by water analysis is greatly confounded by sampling strategy and frequency and provides very little information on pollution pressure occurring through the extended periods of time.

#### 4.1.2. Genetic Diversity and Population Differentiation

The populations Cl and Dp had nearly identically high levels of genetic diversity (Cl: 0.8164, Dp: 0.8065). Similarly high levels of genetic diversity (0.79–0.94) assessed by microsatellite analysis have been reported for other populations of* D. polymorpha* in the literature [23, 48]. The fact that the genetic diversity of the contaminated population was not altered by the WWTP effluent is coherent with the conclusions derived from a meta-analysis across different taxa (plants, invertebrates, and vertebrates) by DiBattista [49]. He found that pollution could both decrease and increase genetic variation in populations (defined as genetic diversity or mean number of alleles per locus) through genetic drift and directional selection on the one hand and increased mutation rate and selection for heterozygotes on the other hand.* Dreissena polymorpha* larvae remain in the plankton up to three weeks or even longer and consequently can be drifted over 300 km before settlement [50, 51]. This specific life history trait makes mutational load as a cause of high genetic diversity highly unlikely. Thus, the high genetic diversity seen in population Dp could have resulted from various selective and stochastic genetic processes. Moreover, decreases in genetic diversity can be diluted by recent migration events, as it was suggested for contamination exposed populations of the arctic amphipod* Orchomenella pinguis* [52] and the Mediterranean mussel* Mytilus galloprovincialis* inhabiting polluted marine ecosystems [53]. The latter study observed higher genetic diversity of mussel populations exposed to environmental pollution, what is in high concordance with our results.

There was no significant genetic differentiation between the two populations Cl and Dp. A high gene flow between the two populations is consistent with their high genetic similarity. In this part of the river Drava, gene flow in the* D. polymorpha* mussels could only result from the downstream swamping of free-swimming larvae in high densities [54]. This passive dispersal mode of larvae strongly prevents genetic population differentiation [55]. As the distance between the two sampling sites Cl and Dp is only about 750 m, it is very likely that the mussels of the Cl and Dp site are offspring of the same mussel populations that inhabited the Čakovec lake or even a location more upstream the river Drava. High levels of gene flow preventing population differentiation are also consistent with the similar high levels of genetic diversity observed in the two populations.

#### 4.1.3. Selection and Genetic Drift

We found a significant deviation from Hardy-Weinberg equilibrium (HWE) for the Dp population, whereas population Cl was in HWE. This deviation observed in Dp was caused by locus C5, which only deviated significantly from HWE and showed a deficit of heterozygotes. While Astanei et al. [23] also reported a deficit of heterozygotes in* D. polymorpha* populations based on microsatellite loci and concluded that it was most likely caused by null alleles, other studies did not find deviations from HWE [48, 56]. Our analyses did not indicate a potential influence of null alleles and suggest that the deviations from HWE at locus C5 must have been caused by another genetic process. Alternatively, it is possible that a locus-specific analysis of population genetic structure reveals novel candidate loci that are the object of selection due to genetic hitchhiking [57, 58]. A linkage of the neutral microsatellite locus C5 to a gene undergoing selection could explain the observed deviation from HWE. We hypothesize that locus C5 is located near a coding gene that is under selection only in the contaminated Dp population, but not in the noncontaminated Cl population. A possible candidate gene could be a gene that is involved in DNA repair mechanisms and/or in antioxidative or detoxification processes (see molecular and cellular level). Our interpretation is corroborated by another* D. polymorpha* population studied by us which inhabits a heavily polluted site in the river Sio, Hungary. This population also showed a deviation from HWE at locus C5 (unpublished data). Other population genetic studies also found a selective effect on a single microsatellite locus that was explained by the genetic hitchhiking model [58, 59]. A genetic linkage mapping of the microsatellite loci for* D. polymorpha* could clarify which coding gene is linked to C5.

The results obtained for age classes also indicated an indirect selection process affecting locus C5. For population Dp, the inbreeding coefficients (*F*_IS_) increased with the age of the mussels and significantly differed from zero for the age classes 2 and 3. This suggests that the proportion of homozygotes increased with the age of the mussels indicating ongoing selection. The increase in homozygosity is reflected by the overall deficit of heterozygotes observed in Dp.

In total, our results derived from microsatellite analysis support hypothesis 1.1 that a genetic bottleneck had affected the Dp population, but not the Cl population. This bottleneck caused random genetic drift. Moreover, we also detected a strong indirect selective effect on locus C5 in the Dp population. The selective process causing this indirect effect on C5 has obviously been acting on the zebra mussels for years, which we demonstrated by the age class analysis.

### 4.2. Field Populations—Molecular and Cellular Level

Hypothesis 1.2 which assumes that the field population Dp should have a higher level of genotoxic effects than the field population Cl was confirmed only by MNT. We observed a significantly higher percentage of micronuclei for Dp than for Cl. This result indicates pollution at the Dp site. The levels of micronucleated hemocytes found for zebra mussels from Dp site are comparable to the levels reported for other polluted sites in the literature [15, 60, 61]. The MNT result is contrary to the Comet assay, in which the Cl and Dp populations did not show significantly different responses. Several studies have also described that a high response seen in MNT is not necessarily reflected at the same intensity in the Comet assay [62, 63]. The following reasons are given for this inconsistency: first, MNT and Comet assay assess different aspects of DNA damage. MNT detects clastogenic and aneugenic effects, while Comet assay measures single- and double-strand breaks, alkali labile sites, DNA-DNA and DNA-protein crosslinks [63, 64]. Second, the level of DNA damage measured by the Comet assay can be lower than the initial level, as it is possible that some of the damage has already been repaired [65]. The MNT only assesses nonrepairable DNA damage and chromosome loss that will persist until apoptosis of the damaged cells. Third (and in connection to the second), acclimatization of mussels inhabiting a contaminated site through activation of the DNA repair mechanism is possible, what could also be facilitated through differential regulation of genes coding for DNA repair, and even genetic selection could have acted in the favor of better adopted genotypes [65–67]. For example, Black et al. [65] carried out a Comet assay for the freshwater bivalve* Anodonta grandis* inhabiting a site contaminated with lead, cadmium, and zinc. They did not find any increased DNA damage in the mussels originating from this site. Mussels of the same species from a noncontaminated site kept in laboratory showed DNA breakage even at low concentrations of lead. The authors concluded that this discrepancy in response in the Comet assay was due to an enhanced DNA repair mechanism of the mussels from the contaminated area.

As the mussels from the Dp site had a low DNA damage in the Comet assay in combination with an increased rate of micronuclei, we hypothesize that they had adapted to the polluted environment by an enhanced DNA repair mechanism and/or antioxidative defense. Inducement of any of these mechanisms is expected to affect the results of the Comet assay stronger than the results of the MNT.

### 4.3. Laboratory Exposure—Molecular and Cellular Level

After exposure to municipal wastewater, the mussels of the Cl population showed a significantly higher level of DNA damage in the Comet assay than the mussels of the Dp population. In contrast, we observed a significantly higher frequency of micronucleated hemocytes in the mussels belonging to the Dp population. These results coincide with our expectation that the two populations Cl and Dp would react differently under altered environmental conditions due to their different genetic constitution (hypothesis 2.1 or 2.2).

Based on the results of the population genetic analysis, the Comet assay, and the MNT carried out for the field populations, we have hypothesized that the mussels of the contaminated Dp site had been acclimatized to the local WWTP effluent discharge by an enhanced DNA repair mechanism and/or enhanced antioxidative mechanism. This possible acclimatization was apparently reflected only in the successful repair of DNA damage revealed by the Comet assay and not in MNT. A more efficient DNA repair system and/or antioxidative mechanisms in the Dp population resulting from acclimatization processes could also explain the differences in responses of the two populations seen in the pollution treatment in the Comet assay. Acclimatization through the DNA repair system and/or antioxidative mechanisms could be concordant with the low level of tail DNA found in the field population Dp, and the significantly lower percentage of tail DNA found in the laboratory Dp population in comparison to the laboratory Cl population in the pollution treatment. This explanation is consistent with our hypothesis 2.1 for the laboratory experiment, where we assumed that population Dp was more resistant to wastewater due to previously acting selection processes in the source habitat. It is also possible that the DNA repair mechanism and/or antioxidative defense were facilitated by differential gene expression, but the relation between these processes is not clear. So far, the role of specific DNA sequences in the repair of DNA damage has not yet been studied in ecotoxicology and should be the focus of future study [2].

Our alternative hypothesis for the laboratory experiment (hypothesis 2.2) was that if the impacted population (Dp) had a decreased genetic diversity due to random genetic drift, it would be affected by wastewater more severely than population Cl. The microsatellite analysis, contrary to hypothesis 2.2, detected no decreased genetic diversity in Dp in comparison to Cl, but we could show that random genetic drift had affected the Dp population. Possibly, genetic drift and resulting decreased population fitness were responsible for the high rate of micronuclei of the Dp population in the laboratory experiment.

## 5. Conclusions

The mussels of the Cl and Dp populations reacted differently to the exposure to wastewater in our laboratory experiment, which was probably due to their differing genetic constitution. This differing constitution was apparently a consequence of the different environmental conditions, to which the mussels had been exposed to in their natural habitat. The combination of the responses of the three biomarkers gave comprehensive information about the impact of both treated and nontreated wastewater on the genetics of zebra mussels at different levels of biological organization. A limitation of our approach is that it is not possible to directly prove the selection of one or several specific genes by the analysis of neutral microsatellite markers. Therefore, future research should answer which genetic process (e.g., genetic selection or plastic response of differential regulation of the DNA repair mechanism) enabled the adaptation or acclimatization to contamination. A challenging perspective could be the widening of our approach to gene expression studies or genetic linkage mapping of the microsatellite loci to coding genes of the model organism* D. polymorpha *[67]. The main advantage of our approach is the possibility to measure effects on genetics at different levels of biological organization (molecular, cellular, and population level) with a time- and cost-effective system owing to the three well-established techniques. We believe that this research is a case study reinforcing the often proposed need to integrate population genetic measures into ecotoxicological investigations [6, 7, 16].

## Figures and Tables

**Table 1:** Primer set used for microsatellite analysis.

Locus	Primer sequence (5′-3′)
A6	**F:** GTT TCT TTG CCG GTC TAA TAA TAG AGT TAA C
	**R: **GTG ATT GTG TAT CTG CTA TAA ACC
B6	**F:** GTT TCT TCG TGT GCT CAT GTT TCC TCC
	**R:** CGT TGT TCA AGC AAT AAG AAA GAC
B9	**F:** GTT TCT TTT GAC AAT ATC CTG TCT AAT G
	**R:** GCG TGT GTT TTT GAA ACG TG
C5	**F: **GTT TCT TGC ACT GTC AAC GTC ACA CTT TTG
	**R: **CCT TGC TAA CAG CTC GGT TGT ATC
Dpol9	**F: M13(-18)**TGG TTG ATG CAG TGA CCC TA
	**R:** TGT CGC TTG ATC CAT GTT TT
Dpol19	**F: M13(-18)**GCA TTC CAT CAA AAA CAC AGA T
	**R:** GAT CAA CAC CAA AGT TCG TTT C

The primers are based on Naish and Boulding [22], Astanei et al. [23], and Thomas et al. [24]. We slightly modified the primer pairs of the loci A6, B6, B9, and C5 (Naish and Boulding [22]; Astanei et al. [23]).

**Table 2:** Results of water and sediment chemical analyses.

Water parameters	Cl2	Dp	WcW	ZaW	Sediment parameters	WcS
pH	8.07	7.74	7.87	7.20	Co (mg/kg)	10.4
O_2_ (mg/L)	8.68	4.62	4.27	NA	As (mg/kg)	<0.05
Cr (*μ*g/L)	<2	<2	<2	19	Cr (mg/kg)	804
Cu (*μ*g/L)	<20	<20	<20	36	Cu (mg/kg)	242
Zn (*μ*g/L)	<20	<20	<20	88	Zn (mg/kg)	1300
Ni (*μ*g/L)	<5	<5	<5	17	Ni (mg/kg)	44.8
Fe (*μ*g/L)	314	126	99.2	1110	Hg (mg/kg)	1.36
Pb (*μ*g/L)	<5	<5	<5	7	Pb (mg/kg)	218
Cd (*μ*g/L)	<1	<1	<1	<1	Cd (mg/kg)	<3
Mn (*μ*g/L)	24.9	38.8	58.5	84	Mo (mg/kg)	42.4
PCB (*μ*g/L)	<0.02	<0.02	<0.02	<2.0	PCB (mg/kg)	<5
Total PAH (*μ*g/L)	<0.005	<0.005	<0.005	0.255	Total PAH (mg/kg)	27.8
					Mineral-oil	
					hydrocarbons (mg/kg)	176.6

Concentrations of abiotic parameters and contaminants of four water samples and one sediment sample are depicted. Cl2: reference site for water analyses in river Drava (Figure 1), Dp: contaminated site in river Drava (Figure 1), WcW: water sample of the wastewater treatment plant effluent channel that flows into the river Drava, ZaW: water sample of municipal wastewater in Zagreb used in the laboratory experiment, and WcS: sediment sample of wastewater treatment plant effluent channel that flows into the river Drava (W.C., Figure 1).

**Table 3:** Locus-specific and overall genetic characteristics of field populations.

Locus	*F*_IS_ (Cl)	*F*_IS_ (Dp)	*H*_*O*_/*H*_*E*_ (Cl)	*H*_*O*_/*H*_*E*_ (Dp)	*F*_ST_ (Cl/Dp)	*A* (Cl)	*A* (Dp)
A6	−0.032	0.014	0.917/0.889	0.896/0.906	0.007	17	15
B6	0.012	0.064	0.917/0.927	0.830/0.886	0.002	18	13
B9	0.106	0.016	0.771/0.862	0.809/0.822	0.006	10	9
C5	0.043	0.167	0.750/0.784	**0.638/0.765**	−0.010	8	6
Dpol9	−0.102	0.092	0.708/0.643	0.600/0.660	−0.008	7	7
Dpol19	−0.102	0.004	0.792/0.784	0.787/0.791	−0.006	9	9
Across all loci	0.007	0.057	0.809/0.815	**0.760/0.807**	−0.001	69	59

Inbreeding coefficients (*F*_IS_), observed and expected heterozygosity (*H*_*O*_/*H*_*E*_) of population Cl and Dp, genetic differentiation (*F*_ST_) between Cl and Dp, and number of alleles (*A*) for Cl and Dp. None of the *F*_ST_ values was significantly different from zero. Significant deficits of heterozygotes from Hardy-Weinberg equilibrium are given in bold. Note: small negative *F*_ST_ values are statistical artefacts and are observed in populations with very low genetic differentiation.

**Table 4:** Analysis of molecular variance (AMOVA) over all six microsatellite loci for field populations.

Source of variation	Sum of squares	Variance component	% of total variation	Fixation index	*P* value
Among groups	2.320	−0.001	−0.060	*F*_CT_ = −0.001	0.556
Among age classes within groups	9.843	−0.002	−0.069	*F*_SC_ = −0.001	0.542
Among individuals within age classes	222.574	0.077	3.172	*F*_IS_ = 0.032	0.044
Within individuals	223.000	2.354	96.957	*F*_IT_ = 0.030	0.043

Total	457.737	2.428			

The results are weighted averages over all six loci for 85 individuals comprising two populations and divided into three age classes. For the group level, the individuals of the three age classes belonging to the same population were pooled. Listed are the source of variation, sum of squared deviations, the variance component estimates, the percentage of total variance, the fixation indices, and the significance of the variance components and of the fixation indices estimated by performing 1023 permutations for the analyses. Note: small negative variance components; % of total variation and fixation indices are statistical artefacts and are observed among entities with very low genetic differentiation (here groups and age classes).

**Table 5:** Locus-by-locus analysis of molecular variance (AMOVA) across both field populations.

Locus	*F*_IS_	*P* value	*F*_SC_	*P* value	*F*_CT_	*P* value	*F*_IT_	*P* value
A6	−0.014	0.720	0.008	0.154	0.005	0.309	−0.002	0.601
B6	0.032	0.211	0.007	0.146	−0.0003	0.535	0.039	0.161
B9	0.076	0.054	−0.022	0.994	0.013	0.106	0.068	0.085
C5	0.092	0.058	0.019	0.069	−0.017	1.000	0.0943	**0.047**
Dpol9	0.006	0.523	−0.019	0.970	−0.002	0.805	−0.015	0.625
Dpol19	−0.001	0.568	−0.003	0.531	−0.005	0.698	−0.009	0.571

Estimates and *P* values of *F*_IS_ (locus-specific inbreeding coefficients), *F*_SC_ (variances among age classes within populations), *F*_CT_ (variances among age classes among populations), and *F*_IT_ (overall fixation indices) for each of the six microsatellite loci. Significant (*P* < 0.05) *P* values are in bold.
